# Histological evidence of chitosan-encapsulated curcumin suppresses heart and kidney damages on streptozotocin-induced type-1 diabetes in mice model

**DOI:** 10.1038/s41598-019-51821-6

**Published:** 2019-10-23

**Authors:** Sabri Sudirman, Ching-Shu Lai, Yi-Ling Yan, Hung-I Yeh, Zwe-Ling Kong

**Affiliations:** 1 Department of Food Science, National Taiwan Ocean University, Keelung City, 202 Taiwan; 2 Department of Seafood Science, National Kaohsiung University of Science and Technology, Kaohsiung, 811 Taiwan; 3 Department of Medicine, Mackay Medical College, New Taipei City, 252 Taiwan

**Keywords:** Experimental models of disease, Immunology

## Abstract

High blood glucose in diabetic patients often causes cardiovascular diseases (CVDs) that threats to human life. Curcumin (Cur) is known as an antioxidant agent, possesses anti-inflammatory activity, and prevents CVDs. However, the clinical application of curcumin was limited due to its low bioavailability. This study aimed to investigate the ameliorative effects of chitosan-encapsulated curcumin (CEC) on heart and kidney damages in streptozotocin-induced type-1 diabetes C57BL/6 mice model. The results showed that Cur- and CEC-treatments downregulated the blood sugar and total cholesterol level as well as enhanced insulin secretion. However, blood pressure, triglycerides content, and very low-density lipoprotein-cholesterol content were not changed. Histochemistry analysis revealed that both curcumin and chitosan-encapsulated curcumin ameliorated cell hypertrophy and nucleus enlargement in the left ventricular of heart and reduced fibrosis in the kidney, especially after the chitosan-encapsulated curcumin treatment. Our study suggested that chitosan can effectively enhance the protective effect of curcumin on the heart and kidney damages in type-1 diabetes mice model.

## Introduction

Diabetes is a chronic disease resulting from either failure of insulin secretion, insulin action, or both. The defect of insulin mechanism leads to hyperglycemia or high blood glucose^1^. The Non-Communicable Diseases (NCD) Risk Factor Collaboration in 2016 reported that the prevalence of adults with diabetes increased from 108 million (1980) to 422 million (2014) in the world^2^. Additionally, according to the International Diabetes Federation (IDF) Atlas guideline report, the number of diabetes is expected to rise to 629 million in 2045^1^. Type-1 diabetes is characterized by autoimmune-mediated pancreatic β-cell results in the deficiency of insulin, whereas type-2 diabetes is peripheral insulin resistance. However, both forms of diabetes is related to elevated inflammation, oxidative stress, cardiovascular diseases (CVDs), and chronic kidney diseases^3^.

Diabetes is also a major risk factor for health-system costs, mortality, and morbidity^2,4–6^. The previous study reported that the mortality prevalence increased in type-1 diabetes^7^. Moreover, it was also associated with heart failure and cardiomyopathy. The risk of heart failure was independently associated with diabetes and it was increased more than 5-folds in woman and 2-folds in men. Also, the prevalence of heart failure in diabetes is 4-times higher than the general population^4^. Type-1 diabetes is also leads to kidney diseases, such as ischemic damage, diabetic nephropathy, and other renal diseases, and it was significantly associated with a reduction of life quality^5,8,9^. Based on these conditions, type-1 diabetes is one of the main global health problems.

Insulin therapy is the most common treatment for type-1 diabetes. However, it does not always provide the metabolic regulation necessary to obviate the disease associated-complications. Therefore, in modern countries, type-1 diabetes treatment was improved by the use of insulin analogs and mechanical technologies, such as insulin pump and continuous blood glucose monitor^10,11^. However, at least 75% of diabetes patients live in low- to middle-income countries and they cannot provide necessary drugs for their treatment^12^. Additionally, some drugs for type-1 diabetes treatment accompanied by side effects for long-term use, such as stroke and severe gastrointestinal diseases^13^. Therefore, the new approaches have emerged for diabetes treatment including a supplement with natural resources, such as fruits, vegetables, and fish oil or through function foods with their content as pharmaceutical or nutraceutical agents, such as polyphenol, flavonoids, alkaloids, and pigments^14^.

Curcumin is an active ingredient which can be extracted from turmeric (*Curcuma longa*). Both turmeric and curcumin have been widely used as food ingredients and also used for disease treatments. Turmeric has been used as a coloring agent in many food industries and medicinal applications such as treatment of inflammations. Curcumins have been emerged as favorable functional food by its potent antioxidant activity and observed in experimental models for some diseases, such as hepatic and pancreatic diseases, arteriosclerosis, diabetic, and colitis disease model^15–18^. Apart from this, the direct application of curcumin was limited due to several factors. The relatively low bioavailability and poor aqueous solubility of curcumin have been reported as major problems. Therefore, various mechanisms have been formulated to enhance curcumin utilization, such as emulsion, encapsulation, and nanoparticle forms^19^. Encapsulations have been used to enhance the bioavailability of poorly soluble drugs. Curcumin was encapsulated by polymers have been reported, including chitosan, polyethylene glycol-poly(ethyleneimine), and oil body encapsulations^19–21^. Chitosan has been reported as an encapsulation agent due to its favorable properties, such as biocompatible, biodegradable, and high drugs loading efficiency. Curcumin encapsulated in chitosan shown the high encapsulation efficiency and small size of the nanoparticles as well as increased bioavailability^21–24^. Therefore, this study aimed to investigate the ameliorative effects of curcumin encapsulated by chitosan (CEC) on heart and kidney damages in streptozotocin-induced type-1 diabetes mice model.

## Results

### Effects of CEC on body weight

**Figure 1:**
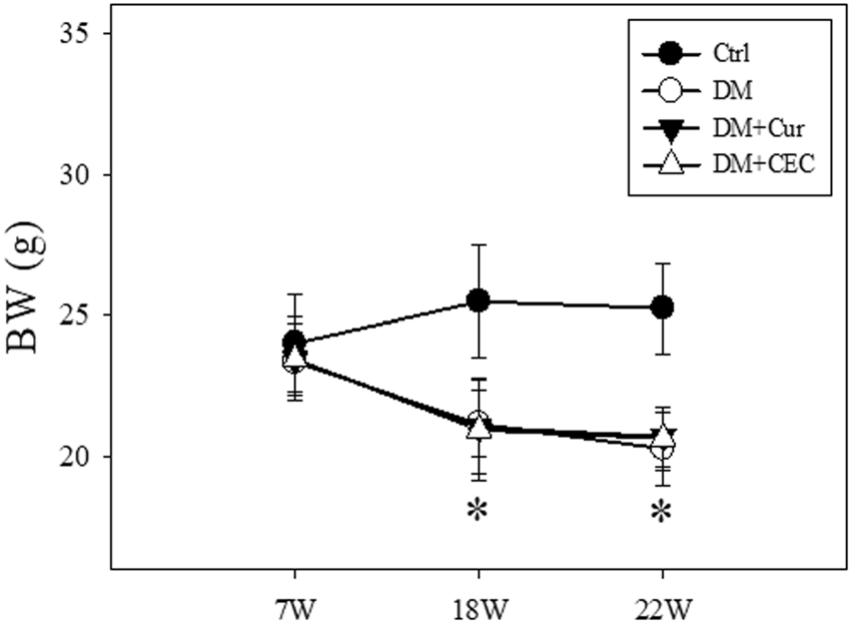
Effect of curcumin and CEC on body weight (BW) after treated for 4 weeks. Data are shown as mean ± S.D. (n = 6). Significant difference: *P < 0.05 vs. Ctrl group as analyzed by Duncan multiple range tests. Ctrl, control group; DM, diabetes without any treatment; DM + Cur, diabetes treated with curcumin (150 mg/kg); DM + CEC, diabetes treated with chitosan-encapsulated curcumin (150 mg/kg).

There were no any significant differences between the initial body weight of mice. The initial body weight of mice no significant differences between all groups. At the week-18 (18 W) of mice’s age, the body weight showed significant difference between the Control (Ctrl) group (25 ± 1.66 g) with another groups (Fig. 1). Treatment with curcumin (Cur) and chitosan-encapsulated curcumin (CEC) for 4 weeks (22 W) suppressed the body weight loss with the body weight of Cur- (21 ± 0.97 g) and CEC-treated (21 ± 1.16 g) groups, whereas these groups were higher than DM group (20 ± 1.29 g).

### Effect of CEC on fasting serum glucose and insulin

**Figure 2:**
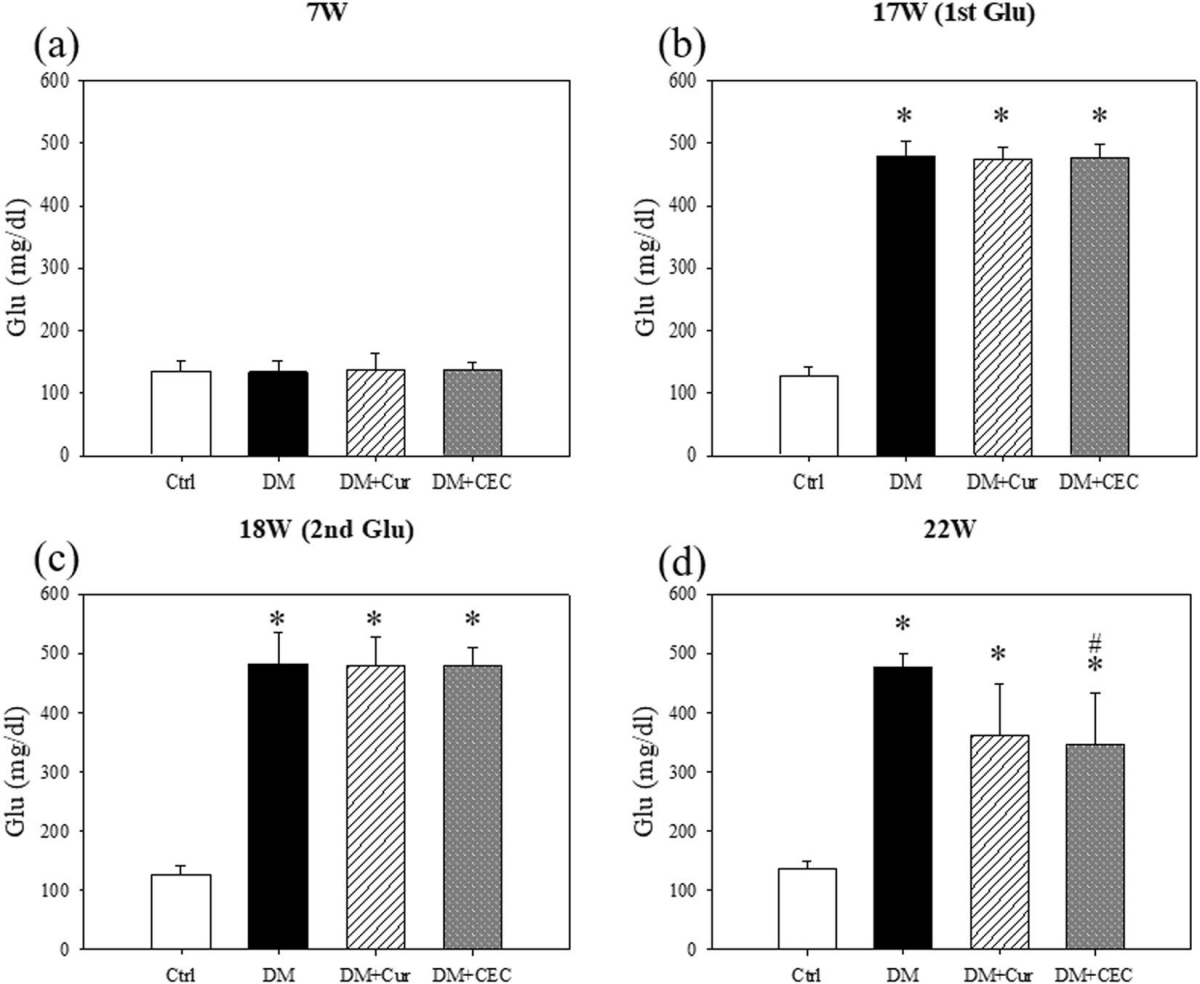
Fasting serum glucose concentration: (**a**) initial glucose level before STZ injection; (**b**,**c**) glucose level after STZ injection at 17 W and 18 W, respectively; (**d**) Glucose level after treatment for 4 weeks with curcumin and CEC. Data are shown as mean ± S.D. (n = 6). Significant difference: *P < 0.05 vs. Ctrl group and ^#^*P* < 0.05 vs. DM group as analyzed by Duncan multiple range tests.

The results showed that the initial fasting blood glucose level was not significantly difference between all groups. However, after intraperitoneally injected with STZ (150 mg/kg), the results showed that the glucose concentration of the STZ injected-groups significant higher when compared to control (Ctrl) group in both 17-week-old and 18-weeks-old (Fig. 2). After treated for 4 weeks (22-weeks-ols) with CEC (DM + CEC, 348 ± 85.21 mg/dL) the glucose concentration was significantly decreased when compared to the untreated-diabetes group (DM, 490 ± 31.98 mg/dL). Whereas, curcumin-treated (DM + Cur) group (363 ± 85.65 mg/dL) showed decreased glucose level, however not significantly different from DM group.

**Figure 3:**
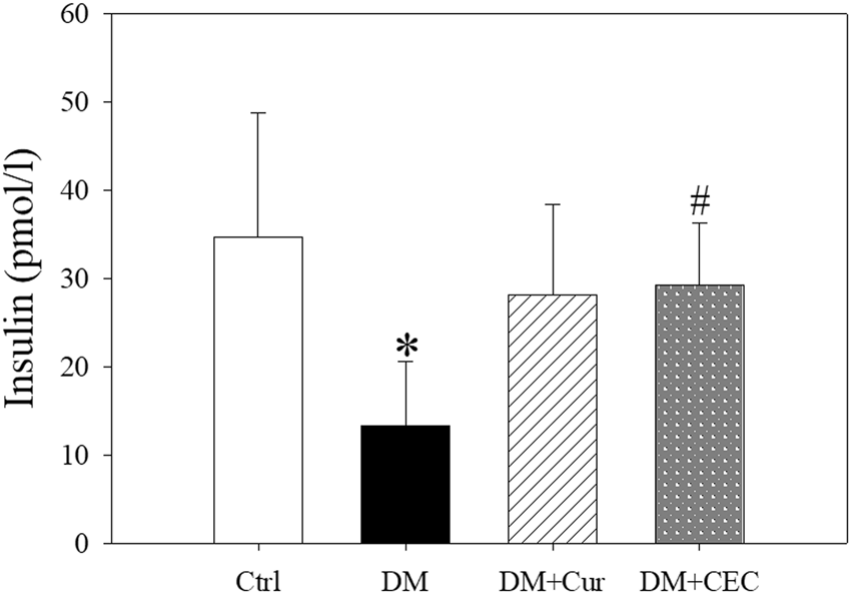
Effect of curcumin and CEC on serum insulin concentration after treatment for 4 weeks. Data are shown as mean ± S.D. (n = 6). Significant difference: *P < 0.05 vs. Ctrl group and ^#^*P* < 0.05 vs. DM group as analyzed by Duncan multiple range tests.

Figure 3 shown that the fasting insulin level of untreated-diabetes (DM) group (13 ± 7.36 pmol/L) significantly lower than control (Ctrl) group (35 ± 14.02 pmol/L). However, treated for 4 weeks by CEC (DM + CEC) group (29 ± 7.14 pmol/L) significantly increased the insulin level when compared to untreated-diabetes (DM) group. Curcumin-treated (DM + Cur) group (28 ± 10.27 pmol/L) also increased the insulin level, however not significantly difference when compared to the DM group.

### Effect of CEC on lipid properties

**Figure 4:**
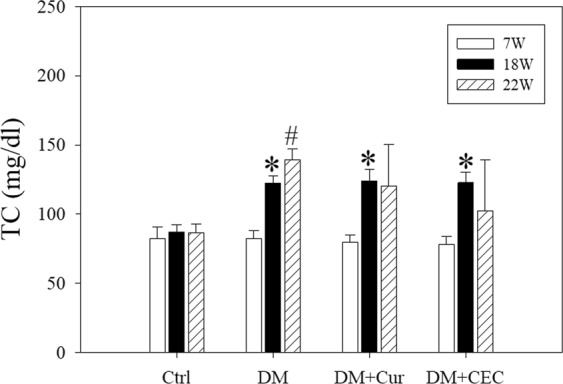
Effect of curcumin and CEC on total cholesterol (TC) concentration after treatment for 4 weeks. Data are shown as mean ± S.D. (n = 6). Significant difference: *P < 0.05 vs. Ctrl group in 18 W and ^#^*P* < 0.05 vs. Ctrl group in 22 W as analyzed by Duncan multiple range tests.

Beside blood glucose and insulin levels, we also observed the total cholesterol (TC), triglycerides (TG), and very low-density lipoprotein-cholesterol (VLDL) to determine the serum lipid properties. At week-18 (18 W), the TC level of all of the diabetes groups significantly higher than the control (Ctrl) group (Fig. 4). After treated with Cur or CEC for 4 weeks, the TC levels were not showed any significant deference when compared to the Ctrl group (87 ± 6.22 mg/dL). Whereas, untreated-diabetes (DM) group (139 ± 8.13 mg/dL) was significantly higher than the Ctrl group. There were no significantly difference between TC level of both Cur and CEC, whereas DM + CEC group (102 ± 36.71 mg/dL) possessed TC level lower than DM + Cur group. For the triglycerides (TG) and VLDL levels, the results showed that there was no significantly difference for all groups **(**Table 1**)**.

**Table 1:** Effect of CEC of total cholesterol, triglyceride and very-low density lipoprotein-cholesterol after treatment for 4 weeks.

Lipid properties	Ctrl	DM	DM + Cur	DM + CEC
TC (mg/dL)	87 ± 6.22	139 ± 8.13*	122 ± 29.91	102 ± 36.71
TG (mg/dL)	69 ± 8.01	67 ± 6.86	71 ± 4.54	73 ± 6.47
VLDL (mg/dL)	14 ± 1.83	14 ± 1.52	14 ± 1.21	15 ± 1.51

Data are shown as mean ± S.D. (n = 6). Significant difference: *P < 0.05 vs. Ctrl group as analyzed by Duncan multiple range tests. TC, total cholesterol; TG, triglyceride; VLDL, very-low density lipoprotein-cholesterol.

### Effect of CEC on hemodynamic of mice

The basic measures of cardiovascular function (hemodynamic), such as systolic blood pressure (SBP), mean blood pressure (MBP), and diastolic blood pressure (DBP) was observed after 4 week’s treatment (Table 2). The results showed that there is no significantly difference in SBP, MBP, and DBP levels for all groups.

**Table 2:** Effect of CEC on hemodynamic data after treatment for 4 weeks.

Parameters (mmHg)	Ctrl	DM	DM + Cur	DM + CEC
SBP	121 ± 5.85	120 ± 3.72	122 ± 6.31	122 ± 1.94
MBP	95 ± 6.15	95 ± 5.17	95 ± 5.35	96 ± 3.66
DBP	82 ± 7.48	82 ± 8.89	82 ± 6.02	83 ± 5.61

Data are shown as mean ± S.D. (n = 6). Significant difference: *P < 0.05 vs. Ctrl group as analyzed by Duncan multiple range tests. DBP, diastolic blood pressure; MBP, mean blood pressure; SBP, systolic blood pressure.

### Effect of CEC on aorta, atrium, and left ventricle of mice histopathology

**Figure 5:**
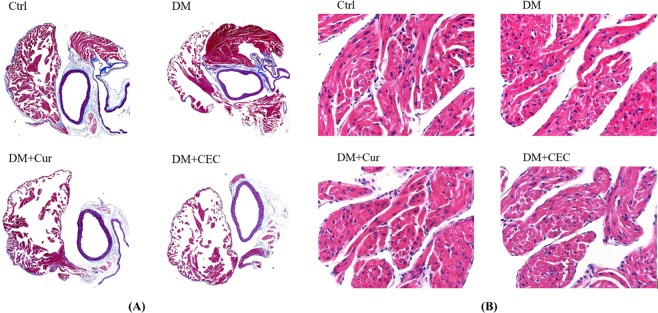
Effect of CEC on heart properties after treatment for 4 weeks. (**A**) Masson’s trichrome stain of the aorta and right atrium, the nuclei are black to brownish black; the cytoplasm is brick red; collagen and mucus bluish green, magnification: 200X; (**B**) H&E stain of the left atrium, Magnification: 400X.

**Figure 6:**
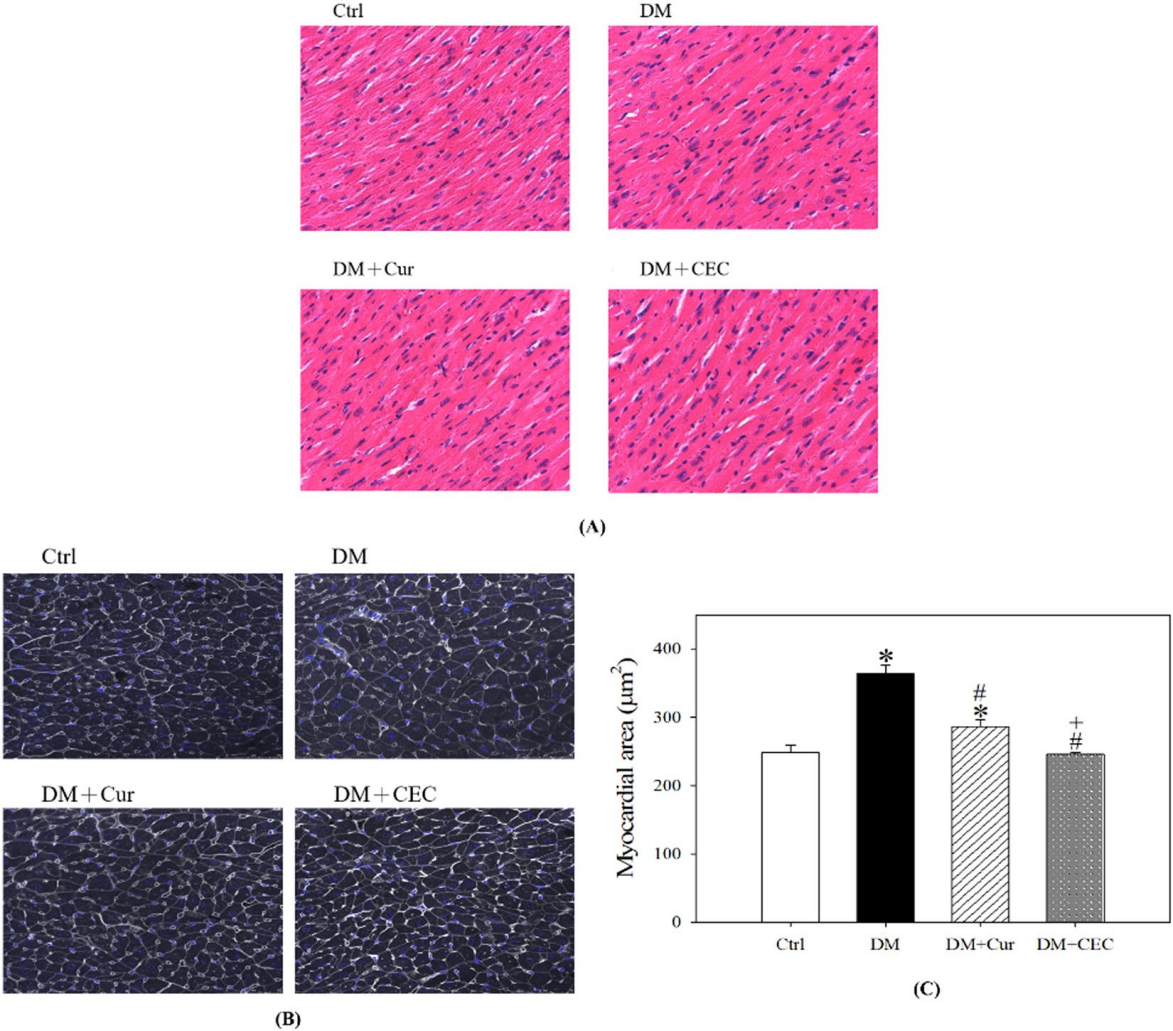
Effect of CEC on the left ventricle of after treatment for 4 weeks. (**A**) H&E stain of the left ventricle, Magnification: 400X; (**B**) Fluorescence microscopy, greys are wheat-germ agglutinin labeled with FITC (WGA-FITC) and blues are the nuclei, Magnification: 400X; (**B**) Analysis of myocardial area in the left ventricle. Data are shown as mean ± S.D. (n = 6). Significant difference: ^*^P < 0.05 vs. Ctrl group, ^#^*P* < 0.05 vs. DM group and ^+^*P* < 0.05 vs. DM + Cur group as analyzed by Duncan multiple range tests.

Masson’s trichrome staining was used to evaluate the aorta and right atrium function, and hematoxylin and eosin (H&E) staining for left atrium and left ventricle. Additionally, WGA-FITC staining also was used to evaluate the myocardial area of left ventricle. According to this staining, there were no any change in collagen composition in the right atrium and does not observed any proliferation of endothelial cells in the aorta blood vessels (Fig. 5A). Additionally, there was no change in cell size in the left atrium (Fig. 5B). However, DM mice showed a nuclear enlargement in the left ventricle (Fig. 6A,B). Treatment with curcumin (DM + Cur) and CEC (DM + CEC) successfully improved the hypertrophy and nucleus size. The myocardial area of DM group (364 ± 74.44 μm^2^) was significantly higher than the Ctrl group (248 ± 40.42 μm^2^) (Fig. 6C). After treated with Cur (DM + Cur, 286 ± 43.83 μm^2^) and CEC (DM + CEC, 246 ± 25.66 μm^2^), the myocardial area was significantly decreased when compared to the DM group. Moreover, the myocardial area of DM + CEC group significantly lower than the DM + Cur group.

### Effect of CEC on kidney properties

**Figure 7:**
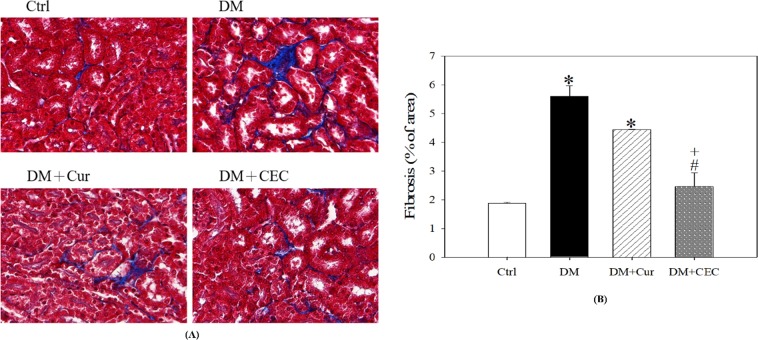
Effect of CEC on the cortex and medulla in the kidney after treatment for 4 weeks. (**A**) Masson’s trichrome stain; the nuclei are black to brownish black, the cytoplasm is brick red, collagen and mucus bluish green, magnification: 400X; (**B**) Analysis of fibrosis cortex and medulla in the kidney. Data are shown as mean ± S.D. (n = 6). Significant difference: ^*^P < 0.05 vs. Ctrl group and ^#^*P* < 0.05 vs. DM group, and ^+^*P* < 0.05 vs. DM + Cur group as analyzed by Duncan multiple range tests.

Masson’s trichrome staining was used to evaluate the kidney histopathology (Fig. 7A). The fibrosis of kidney was significantly increased in DM group (5.60 ± 2.09%) when compared to the control (Ctrl) group (1.88 ± 1.13%) (Fig. 7B). After treated with CEC (DM + CEC, 2.46 ± 0.84%) for 4 weeks, the fibrosis area significantly decreased when compared to the DM group. Moreover, the CEC-treated group also significantly lower fibrosis area when compared to curcumin-treated (DM + Cur, 4.44 ± 1.28%) group. The body urea nitrogen (BUN) was measured to evaluate the kidney function. The results showed that there was no any significant difference in BUN level between all groups. However, the BUN level of DM + Cur (27 ± 4.63 mg/dL) and DM + CEC (25 ± 7.57 mg/dL) groups were lower than the DM group (32 ± 4.97 mg/dL).

## Discussion

Type-1 diabetes has traditionally been associated with weight loss of the patient. Dehydration and muscle breakdown might cause a rapid weight loss in a type-1 diabetes patient^25^. In Fig. 1, the DM mice showed low body weight when compared to all of treatment groups. Both Cur- and CEC-treated mice suppressed the body weight loss. In week-7 (7 W), the mice showed a low glucose level (Fig. 2a). However, the glucose level increased when compared to non-diabetes (control) group after injected by STZ and it was characterized as a diabetes condition (Fig. 2b,c). High blood glucose (hyperglycemia) and low insulin level in blood are also associated with type-1 diabetes condition. Whereas, the high fasting blood glucose (FPG ≥ 126 mg/dL) characterized as diabetes^26^. Although, the fasting blood glucose remains in diabetes condition was significantly decreased after treated with curcumin and chitosan-encapsulated curcumin. (Fig. 2d).

The low insulin level showed in DM group mice (Fig. 3). The insulin level after treated with curcumin and chitosan-encapsulated curcumin was increased. The previous study reported that oral administration of curcumin reversed the hypoinsulinemia and glucose intolerance as well as enhanced the regenerate functional pancreatic islets in STZ-induced Swiss diabetic mice^27^. Additionally, the insulin level of the CEC group was higher than the curcumin-treated group.

The untreated-diabetes mice showed a high total cholesterol level (Fig. 4). After treated with curcumin and chitosan-encapsulated curcumin successfully decreased the total cholesterol (TC) level. The average level of TC in the CEC group was lower than the curcumin group. Whereas, no effect on triglycerides and very low-density lipoprotein-cholesterol level (Table 1). The previous study reported that high insulin level was not only effected to blood glucose level but also associated with cholesterol regulation including its synthesis and absorption^28^. According to our results, the chitosan polymer successfully enhanced the clinical utilities of curcumin in chitosan-encapsulated curcumin form. Additionally, the previous study also reported that nanocurcumin decreased HbA1c and LDL in type-2 diabetes^29^.

Dysfunction and failure organs, such as heart, kidney, nerves, and blood vessel are associated with chronic hyperglycemia. Both of type-1 and type-2 diabetes were associated with cardiovascular diseases and caused disability and death among the patients^6,30^. The hemodynamic diagnosis was used the evaluate the heart performance after treatments. The hemodynamic data showed that the blood pressure both systolic (SBP) and diastolic (DBP), there is no change for all groups after treatment (Table 2). The results showed that their levels remain normal and they are 113–160 mmHg, 81–110 mmHg for SBP and DBP, respectively^31^.

The Masson’s trichrome and hematoxylin-eosin (H&E) stains were used to evaluate the heart and kidney damage caused by diabetes. Based on the H&E staining of the aorta, right, and left atrium of heart, there were no any changed on their function, such as proliferation, hypertrophy, enlargement of the cell, and the collagen structure (Fig. 5). However, hypertrophy and enlargement of the cell were showed by untreated-diabetes mice group on the left ventricle of heart based on the H&E and WGA-FITC staining (Fig. 6). After treated with both curcumin and CEC, the cell size was successfully improved. Additionally, the myocardial area of left ventricle was also improved after treated with both curcumin and CEC. The CEC-treated group showed a low myocardial area when compared than the curcumin-treated group. The previous study reported that cardiomyopathy and heart failure are associated with diabetes condition. A cluster of features including decreased diastolic compliance, myocyte hypertrophy, and interstitial fibrosis are associated with cardiomyopathy in diabetes^32^.

Figure 7A shown the Masson’s trichrome staining of the kidney; and then after analysis, we found that the percentage of fibrosis area of untreated-diabetes group was higher than other groups another group (Fig. 7B). The fibrosis area decreased after treated with both curcumin or chitosan-encapsulated curcumin. Whereas, the fibrosis area of CEC-treated group lower than Cur-treated group. Fibrosis is a characteristic of chronic kidney disease^33^. Diabetes was associated with renal fibrosis and is a major health problem^34^. Additionally, we also determined the body urea nitrogen (BUN). The result showed that there was no statistical difference between each group. However, BUN level decreased after treated with curcumin and CEC. The previous study reported that an increased risk of incident diabetes is associated with high BUN and is associated with failure of kidney function^35^. In this study, we have demonstrated that curcumin and CEC showed ameliorative effect on heart and kidney in STZ-induced diabetic mice. Whereas, CEC possessed more effectively ameliorative effects on myocardial area of left ventricle and fibrosis area of cortex and medulla in kidney.

Type-1 diabetes is characterized by failure of insulin secretion by pancreatic β-cell and resulting in insulin deficiency^3^. Type-1 diabetes in mice model can be induced by intraperitoneal injection of a high dose of streptozotocin^36,37^. Hyperglycemia condition can exert feedback on molecular regulation by the generation of advanced glycation end products (AGEs). Collagen molecules were cross-linked with glycosylated protein, so that collagen cannot be degraded and as resulting in increases fibrosis^38^. The increase of oxidative stress is also associated with cardiomyopathy and leading to DNA damage and increase in AGEs receptor^39^. Therefore, using an antioxidant drug have been shown the beneficial effects of diabetes patient with cardiovascular diseases^40^. Curcumin has been reported to possess strong antioxidant activity and anti-inflammation, including in the case of type-1 diabetes^41,42^. Additionally, curcumin also has been demonstrated its ameliorative effects on the renal injury and diabetes-associated liver disorders in rodent model^43,44^. *In vitro* study, curcumin have been reported for its ameliorative effects on leukocytes infiltration by inhibiting intracellular adhesion molecule-1 expression^45^. Curcumin also inhibited pancreatic leukocytes infiltration in diabetic mice study by reducing proinflammatory cytokines and nuclear factor (NF)-κB activation^46^.

However, the limited application of curcumin such as poor aqueous solubility and low bioavailability have been reported. The previous study reported that nanocurcumin has been showed promising therapeutic enhancement more than curcumin alone^47^. Encapsulation by natural polymers has been used to enhance the bioavailability of poor solubility compounds, such as using chitosan. Chitosan polymer has been reported as an encapsulation agent by its favorable properties, such as biocompatible, biodegradable, and high drugs loading efficiency^19,21^. We used ethyl-acetate and ethanol for the chitosan solvent during the encapsulation process. According to the previous study, ethyl acetate and ethanol showed less toxicity. Ethyl acetate showed low toxicity in mice model with the lethal dose 50 (LD_50_) of orally administrated was higher than 2.0 g/kg/day and ethanol was about 8.3 g/kg^48,49^. Additionally, we also used freeze-drying process to obtain the CEC powder and the previous study reported that very less ethanol residue after freeze-drying process^50^. According to these conditions, we hypothesized that chitosan solvents used in the encapsulation process are safe for oral administration, especially in this model.

Previous study reported that the curcumin ameliorated diabetes models by attenuates tumor necrosis factor (TNF)-α and enhanced enzymatic antioxidant activity. This study also reported that curcumin administration protected pancreatic islet damage in STZ-induced diabetic mice^27^. Curcumin also upregulated nuclear factor erythroid-2-related factor-2 (Nrf-2) and reduced oxidative stress levels in skeletal muscle of high-fat diet-induced oxidative stress and glucose intolerance^51^. Additionally, curcumin reduced segmental sclerosis of glomerular and tubular structure as well as macrophages infiltration in kidneys of diabetic rats by inhibiting nuclear factor-kappa B (NF-𝜅B) activation^52^. Curcumin also improved histological abnormalities and fibrosis of diabetic kidney by inhibiting JNK/NF-kB activation^53^ and also regulates lysosomal enzymes activities (*i.e*. N-acetyl-β-d-glucosaminidase, β-d-glucuronidase) in spleen, liver, and heart of diabetic rats^54^.

Our previous study demonstrated that curcumin was encapsulated by chitosan and silica (SCNP) showed the small particle size (61.7 nm) with an almost spherical shape. This study also showed the effectively anti-oxidative activity on SCNP when compared to curcumin alone^55^. Additionally, the previous studies also reported that curcumin encapsulated in chitosan shown the nano size and high encapsulation efficiency in their forms as well as successfully carried the curcumin in nanoparticle matrix^21–23,56^. And also, chitosan-encapsulated curcumin increased solubility and bioavailability when compared to curcumin alone^24,57^. Based on our results, we found that both curcumin and chitosan-encapsulated curcumin suppressed the heart and kidney damage in type-1 diabetes mice model. Whereas, the chitosan-encapsulated curcumin more effectively to ameliorate the damages.

## Conclusion

Overall, type-1 diabetes successfully induced by intraperitoneal injection of a high dose of streptozotocin. Both curcumin and chitosan-encapsulated curcumin successfully reduced the blood glucose and total cholesterol level as well as ameliorated the insulin level. Whereas, chitosan-encapsulated curcumin possessed more effective effects when compared to the curcumin-treated group, especially in the case of suppressed blood glucose level and increased the insulin level. Chitosan-encapsulated curcumin also more effective ameliorated the myocardial area in the left ventricle and fibrosis area in the kidney. Chitosan successfully enhanced the clinical application of curcumin to suppress the heart and kidney damages based on their morphological evaluations when compared to treated by curcumin alone in type-1 diabetes mice was induced by streptozotocin.

## Methods

### Materials and reagents

Curcumin (NutriPhy) was purchased from Chr. Hansen (Hørsholm, Denmark). This extract contained more than 95% of high-purity curcumin. Chitosan (Chitoligo-C, molecular weight = 120 kDa) was purchased from Lytone Enterprise, Inc. (Taipei, Taiwan) and corn oil was purchased from Yuan Shun Food Co., Ltd. (Yunlin, Taiwan). Formaldehyde solution and streptozotocin were purchased from Sigma-Aldrich (Missouri, USA). The fluorescein wheat germ agglutinin (WGA) was purchased from Vector Laboratories Inc. (California, USA).

### Chitosan-encapsulated curcumin (CEC) preparation

The curcumin (500 mg) was dissolved in 20 mL of ethyl acetate and 20 mL of ethanol. The curcumin solution was mixed with chitosan solution (100 mg of chitosan in 300 mL of distilled water) by a peristaltic pump (0.2 mL/min) and continued with sonication at 20 kHz for 10 min as described by the previous study^58^. The filtrate and residue were separated by using filter paper. The filtrate was evaporated by using vacuum evaporator at 45 °C and dried by a free-drying process to obtained chitosan-encapsulated curcumin (CEC) powder.

### Animal experiment

**Figure 8:**
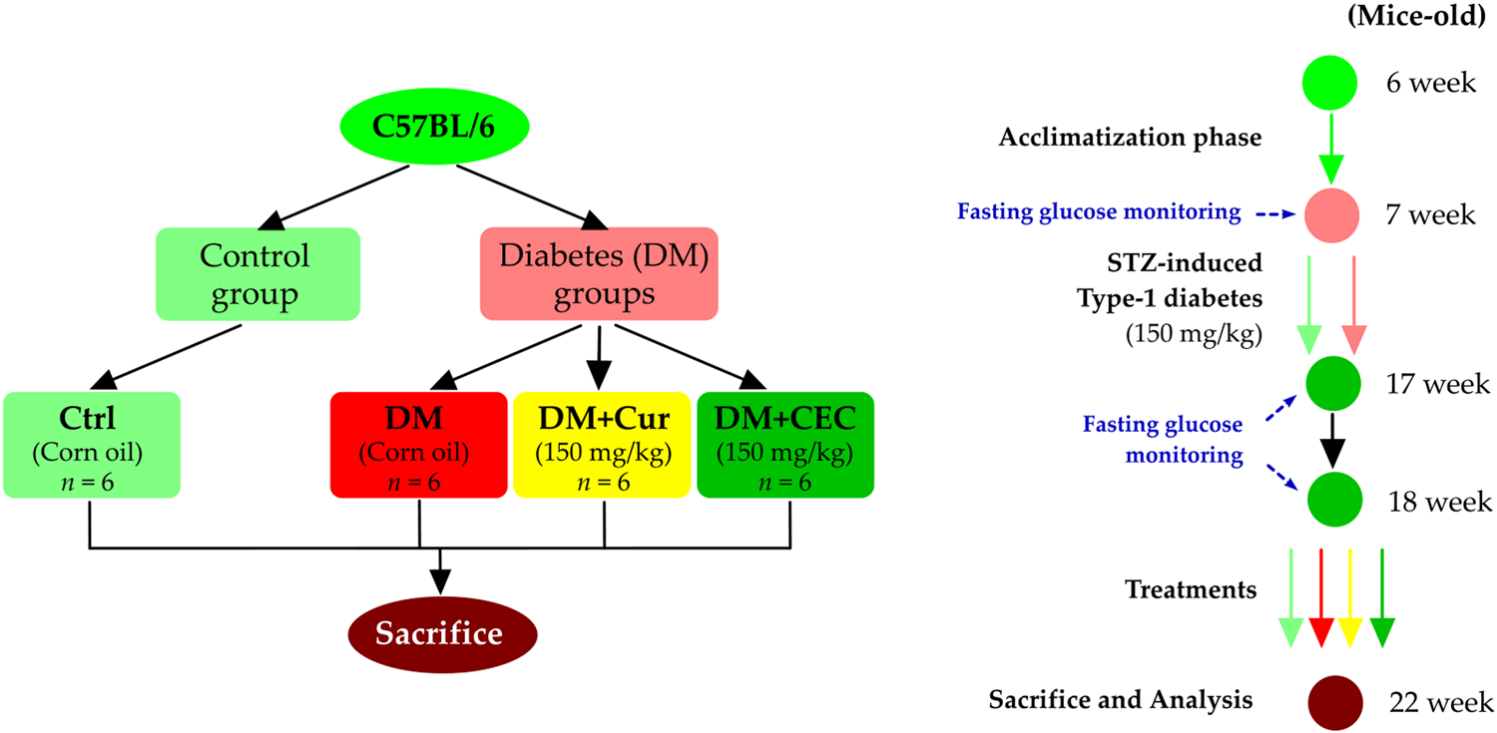
The experimental flowchart of streptozotocin (STZ)-induced type-1 diabetes to induced heart and kidney damages.

Six-week-old male C57BL/6 mice were purchased from the Experimental Animal Center of the National Experimental Research Institute (Yilan, Taiwan). The Institutional Animal Care and Use Committee (IACUC Approval No. 101024) of the National Taiwan Ocean University reviewed and approved all protocols. All experiments were performed in accordance with relevant guidelines and regulations. Briefly, the mice were fed a standard chow-fed diet (Laboratory Animal Diets MF-18) and water *ad libitum*. Mice were housed in a room maintained at 25 ± 2 °C under a 12 h day/night cycle throughout the experiment. Mice were acclimatized for 1 week. After the acclimatization phase, the mice were randomly divided into 4 groups with six mice per group (n = 6). A group was no injected by streptozotocin (STZ) (Control group, Ctrl) and 3 groups were injected by STZ (Diabetes groups, DM) (Fig. 8). Type-1 diabetes was induced by intraperitoneal injection of STZ (150 mg/kg body weight)^36^. The fasting blood glucose of all groups was checked at week-7 (7W) before STZ injection to induce diabetes. The fasting blood glucose also was checked at week-17 (17W) and week-18 (18W) to confirm the diabetes condition. The two groups of diabetic mice were treated by daily oral gavage either with curcumin (DM + Cur, 150 mg/kg) or chitosan-encapsulated curcumin (DM + CEC, 150 mg/kg) and the control (Ctrl) and DM group were also daily orally by corn oil according to previous study^59,60^. The mice were sacrificed on the week-22 (22W) of mice’s age and fasted for 12 h prior to sacrifice. Mice were euthanized by CO_2_ exposure in an empty chamber. The whole blood and organs (heart and kidney) were collected for future analysis.

### Blood sample collection

The blood serum collection method was adapted from previous methods^61^. Briefly, the whole blood of mice was collected using a sterile syringe to collection tubes and allow for clotted for 1 h in room temperature, then centrifuged at 1,000 × g and 4 °C for 15 min to collect the blood serum. The serum was directly used or stored at −80 °C for future analysis.

### Blood pressure measurement

The blood pressure was measured by using the Blood Pressure Monitor (Muromachi MK-2000ST, Japan). This tool was used to measure the blood pressure of small animal such as mice. It possible to measure blood pressure without preheating animals provided that room temperature is at least 23 °C. The measurement was conducted according to protocol manufacture protocol.

### Fasting serum blood glucose, insulin, and lipid properties analysis

The blood glucose, total cholesterol (TC), triglycerides (TG), very-low density lipoprotein-cholesterol (VLDL-C), and body urea nitrogen (BUN) were measured by using blood biochemical analyzer (Biochemistry Analyzer, Kodak Ektachem DT60II, USA). The insulin level was measured by a commercial enzyme-linked immunosorbent assay (ELISA) kit which purchased from Mercodia AB (Cat. No. 10-1247-01, Uppsala, Sweden). All the analyses were performed according to the manufacturer’s protocols.

### Histopathology analysis

The organs were collected at the day of sacrifice. The heart and kidney were fixed in 10% formalin solution. The cortex and medulla in the kidney were using to evaluation kidney histopathology. Then, the organs were embedded with paraffin and cut to slices (5 µm), then stained by hematoxylin-eosin (H&E) and Masson’s trichrome staining^62^. The stained-organs lesion was observed by using upright bright-field system microscopes (Optical Microscope, Nikon Eclipse 80i, USA and TissueFAXS system, TissueGnostics, Austria). The quantification of kidney fibrosis was done by using image analysis software (Image-Pro Plus 6.0, USA). In the case of heart staining, the organ was arrested by using St. Thomas’ Hospital cardioplegic solution number 2 (NaCl 110.0 mM, NaHCO_3_ 10.0 mM, KCl 16.0 mM, MgCl_2_ 16.0 mM, CaCl_2_ 1.2 mM) and delivered to 4 °C prior to fixation to analyze myocardial area using wheat germ agglutinin (WGA) as described by the previous method^63^. The myocardial area of the left ventricle was also evaluated by using FITC-labeled WGA and observed by using a fluorescence microscope (Confocal Spectral Microscope, Leica TCS SP5, Germany).

### Statistical analysis

All data were expressed as mean ± standard deviation (S.D.). Multiple comparisons of different groups were analyzed by Duncan’s test the value of *P* < 0.05 significant level using SPSS 22.0 program.

## Data Availability

All data generated or analyzed during this study are included in this published article.
